# Isolated Pancreatic Metastases of Renal Cell Carcinoma—A Paradigm of a Seed and Soil Mechanism: A Literature Analysis of 1,034 Observations

**DOI:** 10.3389/fonc.2020.00709

**Published:** 2020-05-29

**Authors:** Franz Sellner

**Affiliations:** Surgical Department, Kaiser Franz Josef Hospital, Vienna, Austria

**Keywords:** renal cell carcinoma, renal cell carcinoma metastasis, isolated pancreatic metastases, seed and soil mechanism, treatment results

## Abstract

Previously documented arguments, in favor of the suspected impact of a seed and soil mechanism, in the development and progression of isolated pancreatic metastasis of renal cell carcinomas (isPM) are: (1) uniform and independent from the side of the primary tumor distribution of isPM within the pancreas and, (2) the similar survival rates for singular and multiple isPM. In addition, the present study adds new arguments that further confirm the importance of an seed and soil mechanism in isPM: (1) Within the singular isPM, the size of the metastasis does not affect the overall survival; (2) Within the group of multiple isPMs, the overall survival does not depend on the number of metastases; (3) For synchronous and metachronous isPM, survival rates are also not different, and (4) Within the group of metachronous isPM there is also no correlation between the overall survival and interval until metastases occurs. This unusual ineffectiveness of otherwise known risk factors of solid cancers can be explained plausibly by the hypothesis of a very selective seed and soil mechanism in isPM. It only allows embolized renal carcinoma cells in the pancreas to complete all steps required to grow into clinically manifest metastases. In all other organs, on the other hand, the body is able to eliminate the embolized tumor cells or at least put them into a dormant state for many years. This minimizes the risk of occult micrometastases in distant organs, which could later—after isPM treatment—grow into clinically manifest metastases, so that the prognosis of the isPM is only determined by an adequate therapy of the pancreatic foci, and prognostic factors, such as total tumor burden or interval until the occurrence of the isPM remain ineffective.

## Introduction

The term “seed and soil mechanism” (SSM) coined by Paget back in 1882 (1) concisely describes the particular interaction between embolized tumor cells and potential host organs. After a diffuse, systemic tumor cell dissemination emanating from a primary carcinoma, not all of these cells necessarily mature into manifest metastases. Instead, metastasised tumor cells can only develop into clinically manifest metastases, if the metastasised tumor cells (seed) and cells of the host organ (soil) possess distinct biological properties that exactly match each other. If this is not the case, the metastasised cells will be destroyed. The occurrence of clinically manifest metastases is thus preceded by tumor cell selection.

In human medicine, there are essentially three observations supporting this theory: (1) Paget's main argument came from the clinical observation that individual primary carcinomas—though diffusely spreading their tumor cells through the blood stream—did not metastasise diffusely in all host organs, but apparently had predisposition sites (e.g., breast carcinoma and metastases to the bone). (2) A further support of an SSM theory is the relative resistance of certain individual organs or organ systems, such as muscle or spleen, to metastases. This behavior suggests that local factors that can impede the development of metastases are effective in these organs. (3) Decades later, with the absence of otherwise expected countless lung metastases after peritoneo-venous shunt (2, 3) that was used to treat malignant ascites, the first clinical argument was added.

It was, therefore, surprising to discover (4, 5), that a clearly defined, albeit extremely rare tumor entity exists, whose development and progress can be largely attributed to the hypothesis of an exquisite SSM: isolated pancreatic metastases of renal cell carcinoma (isPM).

Another peculiarity of the isPM, which was already shown in 2006 (6) is that neither singular or multiple occurrence of the isPM, nor a synchronous or a metachronous occurrence of the metastases influenced the treatment outcome. Therefore, the aim of the literature analysis in this study was to investigate further and more extensively this unexpected behavior with regards to a possible involvement of SSM and discuss it in the context of known arguments on the impact of a SSM on isPM.

## Material and Methodology

In the present investigation the term isPM was applied to designate those very rare cases of metastatic renal cell carcinoma (RCC) in which singular or multiple metastases occurred in the pancreas exclusively, both synchronously or metachronously to the primary RCC and definitively or at least over a longer period (>0.5 year).

**Figure 1:**
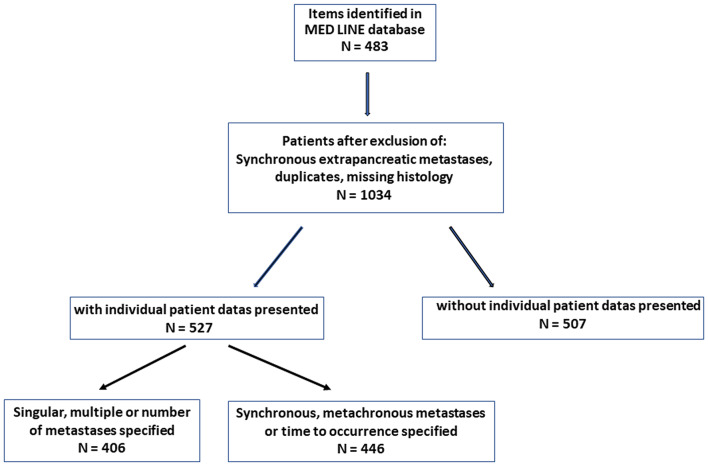
Search and selection strategy.

When presenting previously published arguments (5, 6), the outcomes of previous calculations are reproduced. A literature compilation was prepared for the current investigations on the significance of the time of occurrence and the number and size of isPMs. The observations of synchronous metastases were then compared with metachronous metastases and the singular ones were compared with multiple isPMs. The literature search (Figure 1) was based on the MEDLINE Registry using the key words “renal cell carcinoma and pancreatic metastasis” and covered the period of 67 years, i.e., from 1952 [first description of an isPM (7)] until the end of 2019.

From these publications, only patients with isPMs without metastases in other organs at the time or within 6 months before or after isPM diagnosis were selected provided that these were confirmed by tissue diagnosis. These observations were separated in those with and without individual patient data. For calculations of the influence of solitary vs. multiple metastases only those isPMs were considered in which: (1) the exact number of lesions were specified or (2) those that used wording which was clearly indicative of singularity or multiplicity. In order to separate synchronous and metachronous isPM, analogous selection criteria were applied. Only observations containing unambiguous numerical data were used to investigate the influence of the number and size of metastases. For defining the site of the metastatic lesions, only solitary isPMs that were unequivocally assignable to one specific anatomical part of pancreas (by preoperative imaging, the surgeons report, or the resected specimen) were considered. In the few cases of one single institution repeatedly reporting their isPM observations only, the most detailed report was selected for the analysis. In a retrospective literature review, not every report contained data on all variables evaluated. As a result, the number of calculable observations for subset analyses was reduced. Therefore, the actual number of reports and the references providing information on a given variable are specified.

The literature search revealed a total of 1,034 isPM observations (7–223): 527 casuistic notes were juxtaposed with 507 observations from single center and multicentre studies summaries. All continuous data are presented as means and standard deviations. To determine the influence of RCC side on the site of the metastases within the pancreas—a categorical variable—Fisher's exact test was applied; to determine an equally/unequally distribution of isPM in right side (head) or left side (corpus and cauda) of the pancreas—a dichotomous variable—a non-parametric binominal distribution test was carried out. The differences in survival among subgroups were compared by the log-rank test. A Cox regression analysis was applied to determine the influence of possible risk factors on survival, such as number and diameter of metastasis and the time interval until the occurrence of isPM. *P* < 0.05 was considered statistically significant.

## Results

Arguments for a “seed and soil” mechanism.

### The Metastatic Pathway of isPM

Since the first casuistic notes on isPM, it has been discussed which metastatic mechanism (MM) could lead to the unusual metastatic behavior, with local MM being discussed first. On one hand, a local lymphogenic metastatic MM (28, 72, 85, 90, 143, 165, 177) with a retrograde lymph drainage (158) toward the pancreas as a consequence of a tumorous blockade of retroperitoneal lymph nodes (28, 85, 120, 143, 165) and on the other hand a local venous MM toward the pancreas (28, 72, 85, 90, 165, 177), which is supposed to take place via draining collateral veins of hyper-vascularised tumors (28, 85, 90, 120, 158), or via pre-existing porto-renal anastomoses (90, 158, 224), irrespective of whether renal vein thrombosis was present (28).

The second possible MM is the systemic haematogenic one after intravasation of tumor cells into the circulatory system, which is the case for the majority of organ metastases of solid tumors. The relative importance of the two possible MMs—local or systemic—in the development of isPM can be determined by epidemiological studies. In a systemic haematogenic pathway, the metastases must be diffusely distributed in the pancreas following a uniform distribution with the blood flow, whereas in the local venous/lymphatic pathway, a dependence of the metastasis localization in the pancreas on the side of the primary RCC will occur. Considering that, the left-sided RCC should metastasise more frequently into the nearer corpus and cauda area and right-sided RCC into the nearer caput. Our working group carried out such an epidemiological study for the first time in 2006 on the 236 reported isPM observations (6). Two repeat studies have been subsequently conducted, one on the larger collective of reported isPM observations (*N* = 814) until 2018 (5) and the current study on the 1,034 isPM observations until the end of 2019. Based on the latest research, Table 1 shows the distribution of the reported isPMs between caput pancreatis (48.4%) and corpus and cauda (51.6%), respectively. When this distribution pattern is compared with the volume distribution of the pancreas, 46% in the caput and 54% in the corpus and cauda (225), determined from anatomical studies, no preference for one pancreatic side can be demonstrated (*P* = 0.236). The distribution of isPM in the pancreas is completely uniform and correlates only with the volume distribution, and therefore with the blood flow. Table 2 summarizes the results of the analysis regarding the relationship between the side of the RCC and the distribution of metastases within the pancreas, with the result that the distribution of isPM in the pancreas was independent of the side of the former RCC (*P* = 0.863).

**Table 1:** Distribution of isPM within the pancreas (right side = pancreas head; left side = corpus and cauda pancreatic) (*N* = 256).

**Side**	***N***	****%****
Right side	124	48.4
Left side	132	51.6

**Table 2:** Correlation between the side of the primary renal cell carcinoma and the location of metastases within the pancreas (*N* = 162).

**Localization of the isPM in the pancreas**	**Side of the renal cell carcinoma**		
	**Left**	**Right**	**Both sides**
Caput	47	38	2
Corpus	19	20	1
Cauda	21	13	1
Total	87	71	4

Summing up, these results proved that isPM were evenly distributed over the pancreas and, above all, that there was no dependence on the side of the primary RCC. In the meantime, the latter result was confirmed by a large single institution publications (*N* > 15) (148, 186, 209, 226), which also highlighted the independence of metastasis localization in the pancreas from the primary RCC side. The analysis of the reported isPM thus provided a result that speaks for the dominant importance of a systemic MM. Following the uniform distribution of tumor cells with the blood stream only, this MM will create a uniform distribution of metastasis within the pancreas and also a distribution which is independently from the side of the RCC.

In addition, the mode of distribution of the few metastases recorded (and successfully treated) between primary renal cancer surgery and subsequent pancreatic surgery (25, 27, 61, 68, 69, 72, 87, 105, 129, 136, 139, 140, 150, 160, 166–168, 173, 175, 189, 193, 204, 211, 223) also shed light on the impact of a systemic hematogenous metastatic pathway in isPM. The 78.0% of these 41 metastases were unequivocally of systemic hematogenous origin. Metastases which might have reflected a local tumor cell spread (e.g., retroperitoneal lymph node metastases, recurrent growths in the former surgical field and contralateral kidney or adrenal metastases), by contrast, carried little significance as they were reported only nine (41) times (22.0%) (27, 105, 136, 140, 166, 175, 211). A comparable dominance of the systemic hematogenous pathway was also documented in the reports detailing the fate of patients after successful isPM surgery. Of 116 metachronous metastases (6, 16, 28, 35, 47, 57, 58, 69, 70, 72, 74, 82, 84–86, 90, 94, 108, 112, 118, 123, 126, 128, 136, 139–141, 149, 159, 160, 166, 168, 173, 175, 176, 178, 180, 181, 183, 187, 198, 203, 204, 218) 76.7% were undoubtedly of systemic hematogenous origin and in no more than 23.3% of the cases, a local pathway had to be taken into consideration (72, 84, 108, 139, 140, 160, 166, 175, 180, 181, 183, 187, 198). In sum, for metastases that occurred both before and after pancreatic surgery those attributable to a systemic hematogenous spread predominated. This corroborates with the idea that systemic hematogenous spread plays a considerable role in isPM (4).

However, the established high significance of a systemic haematogenic MP in the occurrence of isPM raises a fundamental question. Why did a systemic MP lead exclusively to metastases in the pancreas, while all other organs remained free of metastases? Considering the small amount of blood flowing through the 120–180 g of pancreatic tissue, it was very unlikely that all embolized tumor cells are transported exclusively into the pancreas by a pure chance. This is even more true if the multiple metastases observed in about 40% of the patients are taken into account (4), which required repeated synchronous or metachronous tumor cell embolisms. The only known mechanism that can explain this, plausibly at present, is the effect of a pronounced SSM, which permits the colonization of metastasised tumor cells and their growth into manifest metastases exclusively in the pancreas, and either definitively prevents them from settling in all other organs or at least causes years of tumor dormancy (227, 228).

### Singular and Multiple isPM, Number and Size of Metastases

**Figure 2:**
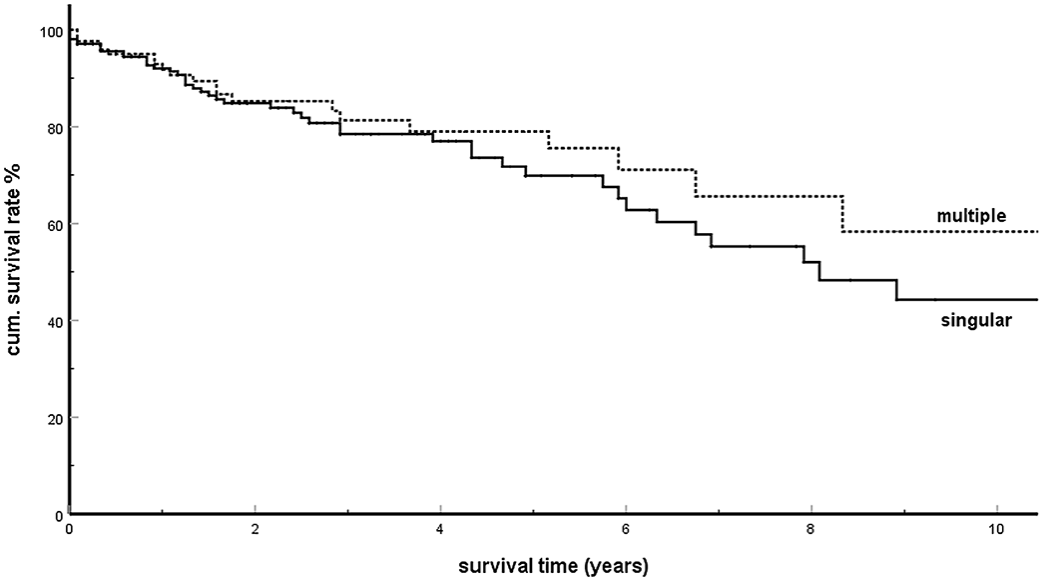
Solitary vs. multiple isPM: Kaplan Meier survival curves (*p* = 0.350).

Already in 2006, in an analysis of 236 isPM observations, our working group assumed (6) that overall survival (OS) of singular and multiple pancreatic metastases in radically operated isPMs were not divergent and, contradicting individual casuistic publications (96, 99, 165, 176), derived from this fact that not only singular but also multiple isPMs had to be radically treated (139). This result was confirmed in the analysis performed in 2018 (5). On the basis of this result, the 1,034 isPM reported so far were analyzed again to investigate whether this unusual result was related to the postulated SSM. The analysis revealed 406 observations in which singular (*N* = 244) or multiple (*N* = 162) occurrences of isPM were undoubtedly mentioned [singular isPMs: (6–8, 10–16, 18, 20–23, 27–30, 32, 38, 39, 41, 43, 44, 46, 47, 49, 51, 52, 54–56, 58, 59, 61, 62, 64, 68–75, 78–80, 82, 85–90, 92, 93, 98, 101, 102, 104, 106–112, 115, 118, 120– 124, 127, 129, 134–137, 140, 141, 146, 149–151, 153, 156, 158–161, 163, 165, 167, 168, 172, 178, 181, 183, 188, 193, 194, 197, 198, 201–204, 206, 208, 210, 212, 214, 216–223); multiple isPMs: (6, 16, 17, 19, 24, 25, 31, 33, 35–37, 40, 42, 45, 48, 53, 57, 63, 65, 67, 68, 70, 72, 74, 76, 77, 80, 81, 83, 85, 86, 88–91, 95, 97, 99, 102–104, 108, 112, 114, 117, 119, 121, 123, 131, 133, 136, 138–140, 145, 147, 149, 150, 152, 160–162, 166, 169, 170, 173, 174, 180, 181, 189, 194, 196, 203–205, 211, 212, 215, 216, 218, 220)]. The comparison of the two groups (Figure 2) further confirmed the similar survival times observed in singular and multiple metastases (*P* = 0.350). This result was confirmed by four single institutions (148, 177, 184, 186), one multicentre report (182), and a literature covering the last 10 years (194) which found that the presence of singular or multiple pancreatic metastases had no significant influence on survival.

In order to further clarify the relationship between tumor volume and survival in isPM, the direct correlation of OS and tumor diameters within the subgroup of singular isPM (*N* = 244) and the correlation of OS and the number of pancreatic metastases within the group of multiple isPM (*N* = 162) was investigated (Table 3). Neither the diameter of the singular nor the number of multiple isPMs proved to be relevant prognostic factors (*p* = 0.423 or 0.754). For “size” of isPM this result is confirmed by two single institution (148, 184) and one multicentre report (182) and a literature review of the last 10 years (194). In summary, as size and number of metastases define the tumor burden both investigations again show that in case of the isPMs the outcome does not depend on the overall tumor burden at the start of therapy.

**Table 3:** Univariate Cox proportional hazards model for overall survival.

**Variable**	***N***	**Mean**	**SD**	**Hazard ratio**	**95% CI**	***p***
Size of metastasis (mm)	125	35.9	20.9	1.009	0.988–1.030	0.423
Number of metastases	83	3.2	1.7	0.944	0.661–1.350	0.754
Time interval (years)	363	10.1	6.3	1.005	0.971–1.040	0.788

*N, number of cases with adequate data; SD, standard deviation of mean*.

To the best of our knowledge, the only explanation for this unusual behavior of isPM with an outcome independent of tumor volume at the start of therapy is a selective SSM, which only allows the growth of metastasised tumor cells to manifest metastasis in the pancreas and completely prevents it in all other organs or at least blocks it for years. So regardless of whether the overall tumor burden and dependent number of metastasized tumor cells are small or great, in case of isPM, the human body can render harmless or eliminate these cells in all organs with the exception of the pancreas. So, the prognosis of the isPM is only determined by adequate therapy of the only remaining pancreatic foci, and the factor “total tumor burden” remains ineffective.

### Synchronous and Metachronous Metastases in isPM

In 2006, our working group pointed out another special feature of isPM (6): the OS was identical for synchronous and metachronous isPM after adequate therapy. This behavior then led to the conclusion that synchronous isPM should also be treated radically. Therefore, for the first time, the striking result of not differing outcomes following treatment for synchronous and metachronous metastases will be analyzed with regards to the effectiveness of an SSM.

**Figure 3:**
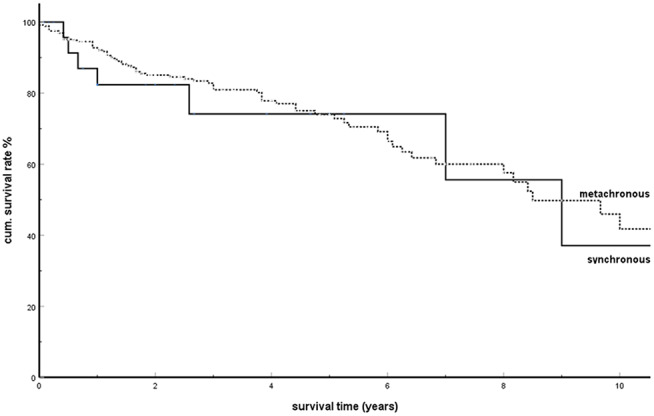
Synchronous vs. metachronous isPM: Kaplan-Meier survival curves (*p* = 0.790).

Among the 1,034 isPM observations, there were 446 in which a synchronous or metachronous occurrence was undoubtedly mentioned. Of these, 30 isPMs were synchronous compared with 416 metachronous ones [synchronous isPMs: (8, 10, 14, 15, 19, 20, 23, 35, 48, 50, 57, 65, 82, 86, 88, 94, 101, 102, 108, 112, 118, 121, 137, 156, 173, 178, 181, 204, 221); metachronous isPMs: (6, 7, 9, 11–13, 16–18, 21, 22, 25–34, 36–47, 49–59, 61, 62, 64, 66–80, 83–93, 95, 97–99, 102–108, 111–115, 117, 119–124, 126–129, 131–136, 138, 140, 141, 143, 145–147, 149–153, 155, 158–162, 165, 167– 170, 172–176, 180, 181, 183, 187–189, 193, 194, 196–198, 201–206, 208, 210–212, 214–218, 220, 222, 223)]. Figure 3 shows the result of the comparison of the cumulative survival rates (SR) between synchronous and metachronous isPMs: with cumulative 5-years SR of 0.741 and 0.740, respectively, the SRs between these two groups do not differ significantly (*P* = 0.790).

**Figure 4:**
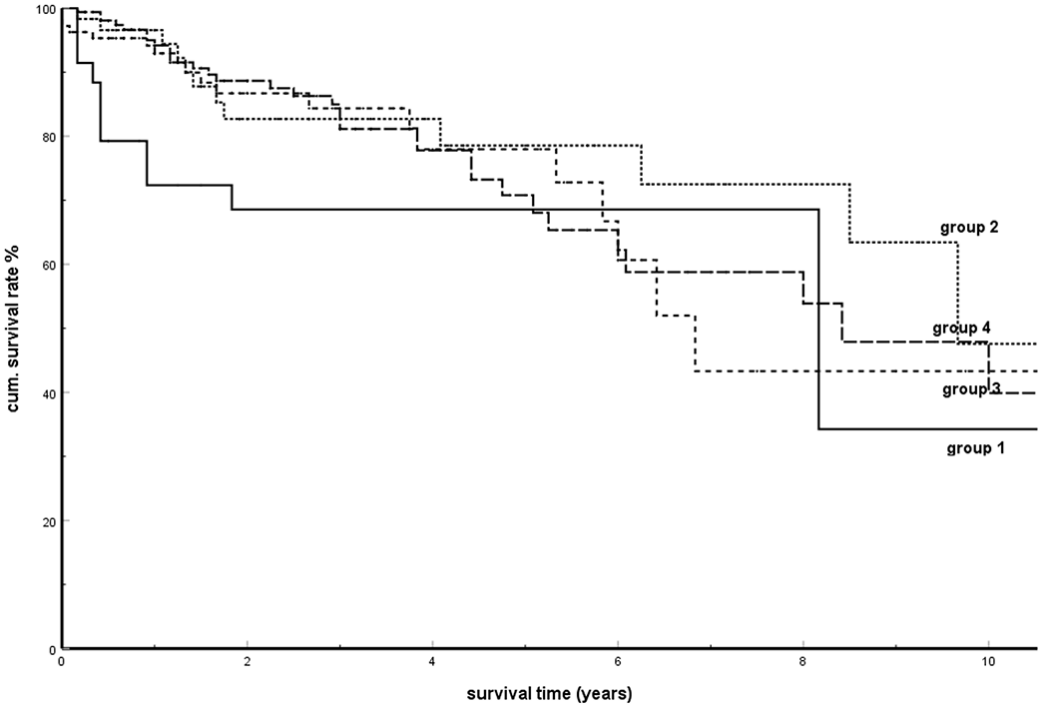
Kaplan-Meier survival curves for metachronous isPM according to interval between tumor nephrectomy and isPM detection (Group 1: <2a; group 2: 3–5a, group 3: 6–10a; group 4: >10a; *p* = 0.339).

In a further study, the SRs were calculated and compared in four subgroups determined by the interval until the occurrence of pancreatic metastases (1. metastases within 2 years; 2. between 3 and 5 years; 3. between 6 and 10 years; and 4. metastases >10 years after the tumor nephrectomy). Figure 4 shows these results. There was no significant difference between the SRs among these groups (*P* = 0.339). The analysis of the influence of the time interval from tumor nephrectomy to the occurrence of metastases in metachronous isPMs on the survival (Table 3) finally provided a consistent supplementary result. The time interval was not detected as a parameter relevant for the prognosis (*P* = 0.788).

In summary, this analysis showed that the factor “time of occurrence of metastases” had no influence on OS both when comparing synchronous with metachronous metastases and within the group of metachronous isPM. Comparable results were reported in single institution (177, 184) and multicentre reports (182), as well as in two literature reviews (142, 194) and in a review of total pancreatectomies in isPM patients (229), which registered no dependence of the results on the interval after a nephrectomy.

According to current knowledge, the unusual behavior of isPM can be indeed explained by an SSM. Regardless of when, depending on the aggressiveness of primary tumor growth, systemic tumor cell dissemination occurs, the organism is able to eliminate these cells in all organs except the pancreas or at least put them into a dormant state for many years, so that the prognosis of isPM is only determined by adequate therapy of the pancreatic foci, and the “interval” factor remains ineffective too.

## Discussion

### Clinical Presentation

The RCC is the ninth most common malignant tumor, with over 330,000 new cases each year (230). At the time of diagnosis, 20–30% of those affected already were in the generalization stage (181, 182, 195), and 15–25% of the curatively operated patients will subsequently develop metastases (136, 158, 182, 190, 231) in lung, bone, liver and brain (139, 157, 158, 176, 190, 195, 200, 232). A special feature of RCC, occurring in about 20%, is a protracted course with long-term phases of low tumor progression or even stability for many years (136, 157, 176, 181, 193, 231, 233). The risk of developing metastases more than 10 years after RCC surgery has been reported to be 6.4 (157) to 11% (232). This group with protracted course also includes the exquisitely rare entity of isPM.

### Treatment Results

**Figure 5:**
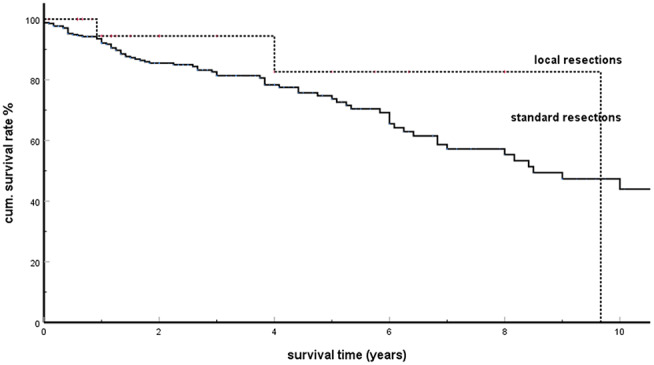
Local resections vs. standard resections in isPM: Kaplan-Meier survival curves (*p* = 0.368).

In addition to the exclusive occurrence of pancreatic metastases over many years, other distinctive features of isPMs are (5, 184, 220) (Table 4) late occurrence of metastases [about 10 years after the operation of the RCC (105, 120, 139, 180, 190)], frequent multiple occurrence (40%), and an unusually favorable prognosis for metastatic surgery with a 5-years survival rate of 73%. Reported 5-years SRs vary from 43 to 100% [Thompson 43% (88), Madkhali 50% (209), Tosoian 52% (184), Bassi 53% (105), Konstandinidis 61% (148), Schwarz 63% (182), Sweeney 65% (234), Strobl 67% (235), Ito 70% (207), Fikatas 71% (195), Chatzizacharias 71% (200), Hung 73% (164), Law 75% (108), Sohn 75% (95), Kimura 77% (177), Yuasa 79% (192), Grippa 80% (122), Zerbi 88% (139), Bahra 100% (132)]. A possible explanation for the unusual favorable treatment results could provide the postulated effect of a specific SSM which is effective in isPM. As this SSM absolutely prevents the settlement of embolized tumor cells in all extrapancreatic sites (or at least forces them into a dormant state), this implicates that the successful resection of isPM constitutes the radical elimination of all active tumor tissue and thus inevitably leads to favorable results (in context with the favorable prognosis of treated isPM, those studies (192, 236, 237) are noteworthy that showed that in diffuse metastasing RCC the concomitant presence of pancreatic metastases generally had a positive prognostic relevance). Depending on the site of isPM in head, body or tail or diffuse distribution, and on the number of metastasis within the pancreas standardized surgical techniques are recommended in form of duodeno-pancreatectomies, distal pancreatectomies, and total pancreatectomies. It is, however, remarkable that the 50 atypical local resections performed, provided a correct R0 resection was performed (51, 70, 72, 105, 109, 113, 121, 139, 140, 143, 147, 160, 166, 177, 181, 182, 186, 194, 209, 222), brought forth survival results which did not differ from standard operative procedures (Figure 5, *P* = 0.368). These results were confirmed by two institutional reports (166, 177) and one literature review of the last decade (194). As standard operative procedures differ from atypical local resections particularly in the greater extent of lymphatic dissection in the former, the identical outcome again demonstrates a minor impact of a lymphogenous tumor cell propagation in isPM. These treatment results emphatically prove that isPM is not a random, first manifestation of a just beginning generalization stage, but an independent, special course of the metastatic renal cell carcinoma (mRCC) caused by an SSM.

**Table 4:** Analysis of literature review of 1,034 isolated pancreatic metastasis from renal cell carcinoma (7–223).

**Variable**	**Data**	**%**
Age (years; *N* = 765)	63.2 (9.7)	
Sex (m:f)	423:359	54:46
Synchronous:metachronous	30:416	7:93
Time to onset (years; *N* = 446)	9.6 (6.7)	
Multiple (*N* = 406)	162	40
Localization (head, body, tail)	124:55:77	48:21:30
Size (mm; *N* = 245)	37.5 (20.8)	
Peripancreat. lymphnodes (*N* = 378)	19	5
Surgery:DP:dP:tP:loc Res^*^ (*N* = 651)	248:244:109:50	38:37:17:8
Histology (clear cell:non-clear cell)	252:5	98:2
Actuarial 5-years survival (*N* = 409)		73

* *DP = duodenopancreatectomy; dp = distal pancreatectomy; tp = total pancreatectomy; loc Res = enucleation and segmentresection. N = number of observations with adequate data (standard deviation of mean)*.

For many years, surgical removal was the only therapeutic option for isPM (6). In the recent decades however, dramatic and encouraging changes have been made regarding the medical treatment of mRCC. Targeted therapies with multi-tyrosine kinase inhibitors, MTOR inhibitors, VEGF inhibitors and immunotherapies proved to be highly effective in mRCC (238–243). The improvement also concerned the results of medical treatment of the few isPM patients reported so far (163, 178, 190, 244–247). Future prospective studies will have to show the significance of surgical or drug therapy, or combinations of both, for respectable isPM (166, 182, 186, 191).

### Metastases Recurrence (Table 5)

**Figure 6:**
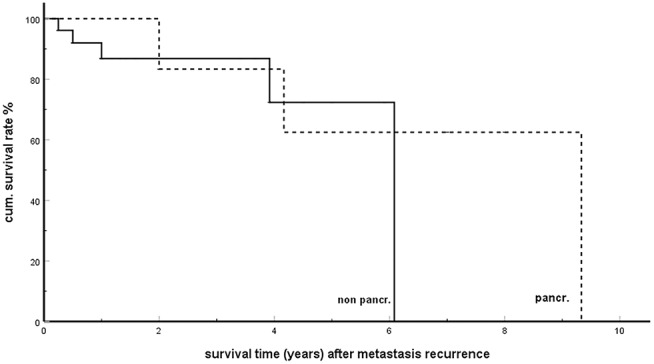
Single organ metastasis recurrence in pancreas (pancr) and in non-pancreatic organs (non-pancr); Kaplan-Meier survival curves (*p* = 0.423).

Out of 265 patients with detailed follow up information in a total of 116 (43.7%) (6, 16, 28, 35, 47, 57, 58, 69, 70, 72, 74, 82, 84–86, 90, 95, 96, 104, 108, 112, 118, 123, 126, 128, 136, 139–141, 149, 159, 160, 166, 168, 173, 175, 176, 178, 180, 181, 183, 187, 198, 203, 204, 218), tumor progression with new distant metastases was observed after a mean disease free interval of 29.3 months (SD 28.1; min 3 max 120 months). The high recurrence rate is confirmed in single- and multicentre reports, reporting on rates of 39–100% [Fikatas 39% (195), Crippa 40% (122), Zerbi 43% (139), Anderson 53% (213), Benhaim 55% (186), Schwarz 60% (182), Santoni 67% (190), and Madkhali 100% (209)]. In no <68.1% of cases, the further recurrence occurred in just one single organ and 21.5% of these were observed as anew isPM in the pancreatic remnant. This result reveals a special feature of isPM: a biological stability of the tumor cells lasting for years and leading, in case of recurrence, to the persistence of the oligometastatic course in a high percentage of cases and even to the occurrence of anew isPM with still favorable prognosis (Figure 6).

**Table 5:** Number of recurrence (N) following treatment of isPM and number of affected organs (cases at risk).

**Recurrence organ sites**	***N***	**%**
All sites	116 (265)	43.7
One organ metastasis	79 (116)	68.1
Pancreas	17	21.5
Non-pancreas	62	78.5
Multiple organs metastases	37 (116)	31.9
2-organs	19	
3-organs	6	
Multiple	12	

The distribution pattern of the subsequently formed distant metastases in different host organ demonstrated a further unusual behavior of the isPM. Though endocrine organs comprise <2% of the volume of the great metastatic organs (liver, bone marrow and brain; Table 6) no <10.3% of the later metastasis (16, 27, 35, 84, 97, 118, 140, 160, 173, 180, 181) were localized in the endocrine system (thyroid 7, adrenal 3 and pituitary gland 2 cases, each). This data suggests a particular preference for endocrine organs as later distant metastases sites in these isPM patients.

**Table 6:** Approximate volumes of different metastatic host organs (ml).

**Endocrine organs**		**Non-endocrine organs**	
Thyreoidea	50	Liver	1,200
Adrenal gland	15	Brain	1,400
Pituitary	2	Bone marrow	2,600
Total	67 (1.2%)		5,200

### Metastatic Mechanism and SSM

Although extensive literature on isPM exists, to our best knowledge, no other investigation has been published which explicitly explores the importance of a possible underlying SSM in this entity.

Our working group hypothesized in 2006 that in isPM “the tumor cells have a high affinity for the pancreas parenchyma” (6). We have since conducted the only known studies in 2018 (4) and 2019 (5) which demonstrated that the pattern of distribution appears to support our hypothesis of a high impact of an SSM in isPM. The more extensive analysis now presented confirms these results and provides additional evidence that there are at least two chains of arguments, which may indicate a high impact of an SSM in isPM: (a) the already suspected mode of distribution of isPM in the pancreas, and (b) newly added the ineffectiveness of risk factors known to influence the prognosis of metastatic solid tumors.

#### Mode of Distribution of isPM and SSM

First of all, the results as stated in section “The metastatic pathway of isPM" once more proved the even distribution of isPM in the pancreas, and in particular, the independence of the metastatic site within the pancreas from the side of the primary RCC. This suggests a high significance of the systemically haematogenic MM in isPM, as the only mechanism that can bring about an even distribution of metastasis in the pancreas and a pattern of distribution which is independent from the side of the RCC. However, if metastases occur exclusively in the pancreas despite systemic cell dissemination, while all other organs remain free of metastases, this can only be explained by a selection process triggered by an SSM. This allows embolized tumor cells to mature into manifest metastases only in the pancreas, while they are not capable of metastasis formation in all other organs. The observed results thus provided a clue for both: a dominant role of a systemic haematogenic MM and a subsequent very effective SSM (4). This only leaves a subordinate significance, if any, for the local lymphatic or venous pathway, which is further underlined by the rare involvement of the lymphatic system in isPM (177). Only in 4.0% of isPM specimen lymph node metastases were present at the time of tumor nephrectomy (57, 143, 163, 186, 187). More importantly, in the rare lymphatic pathway the isolated occurrence of pancreatic metastases also requires an SSM, which allows the settlement of tumor cells and their growth to metastasis exclusively in the pancreas. Only such an elective acting SSM can explain the complete absence of soft tissue metastases in the entire area between the former renal bed and the pancreas and the rare (5.0%) occurrence of peripancreatic lymph node metastases in isPM (105, 108, 116, 139, 148, 166, 177, 182, 184, 186, 188, 189, 209).

The equally low significance of a local venous MM in isPM is supported by the seldom occurrence (10.3%) of a tumorous infiltration of renal veins [Category IIIb; (27, 57, 68, 73, 90, 118, 141, 143)], a prerequisite for a venous flow reversal toward the pancreas. In general, in the case of locally venous MMs, when renal-portal anastomoses lead to the dissemination and colonization of tumor cells into the pancreas, the hepatopedal flow in the portal vein system should transport the blood and the tumor cells it carries in the liver too, with a subsequent increased risk of occurrence of liver metastases. But this was not the case as only in 8.7% of the casuistic reports later on hepatic metastasis were reported (6, 16, 70, 74, 85, 86, 90, 123, 139, 140, 159, 160, 173, 181). The successful colonization in the pancreas with simultaneous absence of liver metastases can again only be explained by an SSM which prevents the settlement of embolized tumor cells in the liver.

In connection with the organotropy in metastatic formation in isPM, it is worth recalling that the pancreas itself is composed of two organ components: a large exocrine and a small endocrine one. At the moment, it remains unknown whether metastatic settlement in isPM starts in the exocrine or endocrine tissue or in both. In this context the above-mentioned observation is at least remarkable, that in instances of single organ metastatic recurrence an increased number of metastasis in endocrine organs was demonstrated (10.3%).

#### Ineffectiveness of Risk Factors

Secondly, an equally important result, the analysis showed that isPMs, too, were characterized by the peculiarity that the risk factors, such as singular/multiple metastases, number of metastases, metastasis size, and synchronous/metachronous metastases and interval from RCC surgery to isPM occurrence remained ineffective. This behavior is surprising, since it is generally applicable in metastatic surgery of solid tumors that the treatment result is influenced by the initial tumor burden (248–250). For mRCC, such studies have also been presented (though without mentioning if isPM observations were included), which unanimously confirmed that the initial total tumor burden was a negative prognostic factor (231, 251–253). Metastases surgery also proved that the time of occurrence of metastases was a prognostic factor, e.g., in liver metastases surgery of colorectal cancer (248, 254, 255), gastric cancer (256), and breast cancer (257). After all, these parameters were also risk factors proven in RCC surgery (232, 233, 258–261), as well as in RCC metastases surgery (262–268), although the significance of these factors varied greatly depending on individual properties of primary RCC and metastasis localization and not all factors were equally effective for every metastatic localization. It should be noted, however, that generally these risk factors are only an expression of the magnitude of the risk that after pancreatic metastasis treatment from occult micro metastases in other organs, a generalization stage will result. It is therefore more remarkable that, as a special feature of isPM, the above-mentioned volume and interval dependent risk factors had no influence on the prognosis of isPM. To the best of our knowledge, this feature of isPM can currently only be explained by an SSM which permitted the growth of metastasised tumor cells to manifest metastasis exclusively in the pancreas and definitely, prevented it in all other organs or at least blocks it for years. The effect of such an SSM causes, that in isPM (almost) all extra pancreatic tumor cells were successfully eliminated or at least kept in a dormant state for years by the host organism. So, regardless of whether a singular or multiple systemic tumor cell dissemination occurs from the primary tumor, regardless of whether a further tumor cell dissemination occurs from singular or multiple metastases, from synchronous or metachronous metastases, or from large or small metastases, the human body is able to eliminate—or at least render harmless—these cells in all organs with the exception of the pancreas. This minimizes the risk of extra pancreatic-located distant metastases so that risk factors reflecting the probability of the later occurrence of distant metastases must remain without influence on survival. Thus, the prognosis of isPM is in an unusual way determined only by adequate therapy of the pancreatic foci and the otherwise effective risk factors “tumor burden” and “interval” must remain ineffective.

So, summing up these results, the mode of distribution and the ineffectiveness of risk factors reveal two argumentations but also the unexpected positive survival rates and the high percentage of single organ metastases or even anew isPM in recurrent disease are observations that can be best explained and made plausible by the effect of an SSM. Together these findings therefore support the hypothesis of a high impact of an SSM in isPM. They further provide evidence that in isPMs, for the first time in human medicine, a clearly defined tumor entity is identified the occurrence and course of which, is exclusively triggered by an SSM making isPM to a paradigm of an SSM.

### Pathomechanisms Leading to isPM

The biochemical mechanism which causes the only occurrence of pancreatic metastases in isPM is unknown as no such studies have been carried out due to the exquisite rarity of isPM (4). However, the RCC as such has been the subject of biochemical investigations that allow conclusions to be drawn by analogy. The effect of an SSM suggests that primary carcinomas which generate a large number of differently equipped tumor cells will gain an advantage in metastasis settlement, as this increases the probability that a cell capable of metastasis in the respective target organ will reach it. The RCC is characterized by a large heterogeneity (238, 269, 270), for which also the variety of miRNA with altered expression behaviors is responsible, which becomes effective in the RCC. miRNAs control the metastasis behavior through their ability to inhibit numerous target genes involved in different steps of the metastatic cascade, e.g., epithelial-mesenchymal transition (271–274), migration (271, 275–279), and metastasis settlement (280–284). Variable interactions of all these miRNAs in various tumor cells bring about manifold different capabilities for metastasis which increases the odds that one of the embolized tumor cell exactly “fits” the properties of the target organ (5). Studies have also shown that the miRNA profile of the metastatic RRC differed from the profile of the non-metastatic RRC (285, 286), and the miRNA profile in metastases also differed depending on the location of the metastases in the lungs, bones or brain (287). These observations suggested that cell selection influenced by the mRNA profile during metastasis formation was involved.

In general, organotropy can always be expected if the early metastasis phase involved steps that require the successful interaction of tumor cell and host organ properties. In literature, mechanisms have already been identified that can generally be held responsible for organotropy in metastasis formation, and whose involvement in isPM seems at least possible: (a) the interaction of a chemokine receptor on the tumor cell surface and a suitable ligand in the host organ (5). This interaction is a necessary prerequisite for the activation of numerous signal transducing pathways, which are critical in tumor cell proliferation, migration, angiogenesis, invasion and proliferation (288, 289). As the chemokine receptor equipment is tumor cell specific and the type and level of the ligand is organ-specific, successful interaction will only take place in those tissues where receptors and ligands fit exactly together (288, 290, 291). Breast cancer e.g., was found to express the chemokine receptors CXCR4 and CCR7 at high levels. The corresponding ligands CXCL12 and CCL21 are present at elevated levels in lymph nodes, lung, liver and bone marrow—preferred distant metastatic sites of breast cancer (288, 290). (b) The formation of a pre-metastatic niche (PMN) (290–296). It is the result of the ability of respective solid tumors, already before tumor cell embolization occurs, to weaken defense mechanisms which are directed against tumor cells in potential host organs. By disturbing humoral and cellular defense mechanisms in host organs, the subsequent tumor cell embolization and colonization can take place successfully and metastases can form in this organ. The formation of a PMN requires the interaction of three components: primary tumor derived components (273, 296, 297), tumor mobilized bone marrow derived cells (298) and organ components of the future host organ (292, 294, 299, 300). In pre-metastatic niche formation, the capabilities of tumor cells and host organs are involved, this causes organotropy in niche formation (294). The fundamental ability of the RCC to form a PMN is documented by proof of a PMN in the lung (299). The ability to form a PMN in the pancreas, however, is so far not documented for RCC. (c) A different immunoediting in various host organs. First, evidence of the importance of immune defense in RCC was provided by observations of spontaneous regression in metastatic RCC, which were interpreted as a result of enhanced immune defense (69, 301, 302). The recent, successful introduction of immune modulating treatment with immune checkpoint inhibitors, such as Anti PD-1 (Nivolumab) or Anti CTLA-4 (Ipilimumab) has impressively demonstrated the high importance of immunoediting in mRCC (239, 241, 301, 303, 304). However, since there are currently no detailed studies of the rare isPM, all considerations regarding the significance of immunoediting (228) in isPM that is either generally altered or only disturbed in the pancreas must remain speculative at present. Although this is quite conceivable, if one regards the aforementioned differentiated miRNA profile in different metastatic organs as a consequence of an immune-dependent selection process. The peculiarity of isPM would then be that in all host organs, except the pancreas, immune-surveillance detects and correctly eliminates the metastasised tumor cells by natural tumor specific T-cell mediated immune response or keeps them in a dormant “equilibrium” state (228). In the pancreas, however, an immunosuppression is present which enables the carcinomas cells to evade immune control and to mature to manifest metastasis. IsPM would thus represent a “single organ deficiency of immune response.”

### Limitations of the Study

Potential weaknesses of the presented literature analysis were related to the retrospective character of casuistic reports and a bias in the published casuistic reports cannot be excluded on principle. This methodical limitation is however, at least in part compensated by the confirmation of some results by large single and multicenter reports in the last years.

## Conclusion

The hypothesis of a strongly and selectively acting “seed and soil” mechanism can not only be explained by the analysis of the pattern of distribution of isPM, but also by studies on the significance of risk factors after therapy, revealing the peculiarities of isPM. This enables embolized renal carcinoma cells exclusively in the pancreas to complete all the steps that are required for growth to manifest metastases without disturbances, while in all other organs the metastasised carcinoma cells are prevented from colonization (290). According to this hypothesis, isPM is a tumor entity with model character that suggests that the occurrence of manifest metastases is preceded by several multi-step, cascade-like biological processes, which require the exact matching of properties of the tumor cell with those of the host organ (287, 290, 291, 305). Even the interruption of one single step can irreversibly disrupt this “colonization” process (3, 306). This susceptibility (291) of the early metastasis process to disruption opens up the chance for the host organism to prevent embolized tumor cells from growing into metastases. With the isPM, this blockade obviously works successfully in all organs except the pancreas. Therefore, with the isPM, a clearly defined tumor entity is given in human medicine, which is characterized to a large extent by an SSM. The uniform and stable clinical course of the isPM also suggests that the isPM is based on uniform pathogenic mechanisms that remain constant for years. This indicates that biochemical investigations would be meaningful to examine the mechanism leading to the exclusive occurrence of metastases in the pancreas and their absence in all other organs. This could help to shed further light upon the complex metastasis process, which is a prerequisite for the development of therapeutics that once might help to hamper the metastasis process (307).

## Author Contributions

The author confirms being the sole contributor of this work and has approved it for publication.

## Conflict of Interest

The author declares that the research was conducted in the absence of any commercial or financial relationships that could be construed as a potential conflict of interest.
